# A Critical Analysis of Purchasing Arrangements in Kenya: The Case of the National Hospital Insurance Fund

**DOI:** 10.15171/ijhpm.2017.81

**Published:** 2017-07-18

**Authors:** Kenneth Munge, Stephen Mulupi, Edwine W. Barasa, Jane Chuma

**Affiliations:** ^1^Health Economics Research Unit, KEMRI Wellcome Trust Research Programme, Nairobi, Kenya.; ^2^Kenya Country Office, The World Bank, Nairobi, Kenya.

**Keywords:** Strategic Purchasing, Universal Health Coverage (UHC), Low- and Middle-Income Countries (LMICs), Health Financing, Social Health Insurance, Financial Protection, Kenya

## Abstract

**Background:** Purchasing refers to the process by which pooled funds are paid to providers in order to deliver a set of health care interventions. Very little is known about purchasing arrangements in low- and middle-income countries (LMICs), and certainly not in Kenya. This study aimed to critically analyse purchasing arrangements in Kenya, using the National Hospital Insurance Fund (NHIF) as a case study.

**Methods:** We applied a principal-agent relationship framework, which identifies three pairs of principal-agent relationships (government-purchaser, purchaser-provider, and citizen-purchaser) and specific actions required within them to achieve strategic purchasing. A qualitative case study approach was applied. Data were collected through document reviews (statutes, policy and regulatory documents) and in-depth interviews (n=62) with key informants including NHIF officials, Ministry of Health (MoH) officials, insurance industry actors, and health service providers. Documents were summarised using standardised forms. Interviews were recorded, transcribed verbatim, and analysed using a thematic framework approach.

**Results:** The regulatory and policy framework for strategic purchasing in Kenya was weak and there was no clear accountability mechanism between the NHIF and the MoH. Accountability mechanisms within the NHIF have developed over time, but these emphasized financial performance over other aspects of purchasing. The processes for contracting, monitoring, and paying providers do not promote equity, quality, and efficiency. This was partly due to geographical distribution of providers, but also due to limited capacity within the NHIF. There are some mechanisms for assessing needs, preferences, and values to inform design of the benefit package, and while channels to engage beneficiaries exist, they do not always function appropriately and awareness of these channels to the beneficiaries is limited.

**Conclusion:** Addressing the gaps in the NHIF’s purchasing performance requires a number of approaches. Critically, there is a need for the government through the MoH to embrace its stewardship role in health, while recognizing the multiplicity of actors given Kenya’s devolved context. Relatively recent decentralisation reforms present an opportunity that should be grasped to rewrite the contract between the government, the NHIF and Kenyans in the pursuit of universal health coverage (UHC).

## Background

Universal health coverage (UHC) - a situation where every citizen has access to needed services, of good quality, without getting into financial ruin or impoverishment - is the leading global health agenda of the new century.^1^ UHC’s objectives of utilisation based on need, financial risk protection and access to quality health services are largely more relevant to low- and middle-income countries (LMICs), although high income countries are also continuously looking for ways to expand coverage to their populations.^2,3^ Several LMICs are reforming their health systems for UHC. These reforms include a greater emphasis on generating additional revenue, particularly through domestic resources, moving away from out-of-pocket payments towards prepayment of health funds through a combination of tax funding and health insurance, and minimizing fragmentation in pooling.^4-6^

Purchasing, one of the financing health functions (the others being revenue generation, and pooling), can be a major cause of inefficiencies, inequities, and can undermine access to quality services.^1^ Purchasing refers to the process by which pooled funds are paid to providers in order to deliver a set of healthcare interventions.^1^ It involves three actions: (1) selecting services and interventions to be purchased, (2) selecting service providers, and (3) determining the contractual and payment arrangements between the purchaser and providers.^7^ Purchasing can be passive or strategic. Strategic purchasing involves a continuous search for the best ways to maximise health system performance, by deciding which interventions should be purchased, how and from what providers, while passive purchasing implies following a pre-determined budget or simply paying bills when presented.^7-10^ There is evidence of a global move towards strategic purchasing.^9^ Experiences from LMICs include the move towards an explicit set of prioritised entitlements that citizens can expect to receive,^11^ the use of contracts and the inclusion of non-state providers of health services,^12^ the introduction of a mix and a variety of provider payment mechanisms,^13,14^ and a movement towards addressing the organisational structure and capacity of purchasing organisations.^15,16^

Purchasing in the Kenyan health sector is performed through three mechanisms. Under the first mechanism, national and county governments purchase healthcare services from public healthcare services that they own. Specifically, the national Ministry of Health (MoH) owns three tertiary care hospitals and pays for services through global budgets. County governments own primary and secondary care health facilities and pay for these services through line budgets, health worker salaries and supply of commodities. Under the second mechanism, the National Hospital Insurance Fund (NHIF), Kenya’s social health insurer, contracts both public and private healthcare facilities to provide services to registered members. The final mechanism is utilised by private and community-based health insurance (CBHI) schemes, which contract public and private health service providers to provide services to members. These mechanisms have been labelled in literature as public integrated, public contract and private contract models respectively.^17^

This study is part of a larger study that assessed each of the three main purchasing arrangements in Kenya. In this paper, we focus our attention on critically analysing the purchasing arrangements of the NHIF. Established in 1966 as a department within the MoH, the NHIF is a semi-autonomous government agency. Its mandate was to provide contribution-based insurance services initially for those in the formal sector and later for those in the informal sector.^18^ A revision of the law in 1998, established the NHIF as a state corporation, affirmed its mandate to provide coverage for the whole population, instituted a governance structure, established new contribution rates and specified means for provider selection and contracting.^19^ Membership to the NHIF is compulsory for all formal sector workers, and voluntary for the informal sector. The NHIF covers about 15% of the Kenyan population, approximately 88.4% of all persons with health insurance in Kenya.^20^ The government of Kenya has made a specific policy decision to, among others, expand health insurance coverage among the population through the NHIF as a means to achieving UHC.^21^ The centrality of the NHIF to proposed and continuing health financing reforms in Kenya makes its purchasing practices essential to the attainment of UHC and therefore an important subject for critical examination.^22,23^ Moreover, the NHIF may offer lessons for a broad variety of settings that are pursuing UHC using a public contributory health insurance mechanism.

## Methods

### Study Setting

Kenya is a lower-middle income country^24^ whose health system is organized around two major administrative levels: national and county. The national government has policy and regulatory roles, while the 47 county governments have healthcare service provision roles.^25^ Healthcare service provision is pluralistic with an almost equal share of private and public providers.^26^ The health system is organized in tiers from community, primary, secondary and tertiary care.^27^ All health facilities should provide services based on the Kenya Essential Package for Health (KEPH), a comprehensive life cycle and level of care based description of health services that the country aspires to deliver to all its citizens.^28^ According to the Kenya National Health Accounts 2012-2013, the main sources of health funding are government (31%), private (40%) and donor (25%) expenditure.^29^ Out-of-pocket payments constituted 32% of total health expenditure (THE).^29^ In terms of shares of non-capital (current) health expenditure (CHE), the main purchasers of health services were the MoH (managing 32% of CHE), household (29% of CHE with 80% spent through out of pocket payments), donors (19%), private health insurers (9%) and the NHIF (5%).^29^ Though these figures precede the devolution of health services to county governments, more up-to-date figures are unavailable. However, in the absence of significant resource generation and pooling reforms, government likely remains a major purchaser of health services. The NHIF is financed through premium contributions to the national scheme from about 5 million registered members and from general government revenues which provided full premium subsidies for 3500 elderly persons in 2015.^30^ The NHIF also manages a special fund for civil servants, police and the Kenya Defence Forces, the Civil Servants and Disciplined Forces Medical Scheme, and a full-premium subsidy program for the poor, the Health Insurance Subsidy Program.^31^ The latter covers about 641 688 individuals across the country.^32^

### Study Design and Data Collection

We employed a qualitative case study approach, with the NHIF as the study case. Data were collected through in-depth interviews (n = 62), and document reviews (statutes, policy and regulatory documents). We purposively sampled persons with knowledge of health financing and purchasing arrangements in Kenya for in-depth interviews. These included officials of the MoH, the NHIF, community-based and private health insurance, health service providers, regulatory authorities and insurance industry associations. The selection of officials of community-based and private health insurance (CBHI and PHI respectively) was because of their relationship with the NHIF. CBHI are community owned and managed organisations that provide insurance at village level though with limited mostly rural coverage comprising <1% of the insured population.^33^ CBHI act as agents selling NHIF insurance as stand-alone or co-packaged products.^34-36^ CBHI officials also provided a window into the relationship between the NHIF and citizens. PHI provide voluntary insurance that is complementary (top-up) for those mandated to be NHIF members (all formal sector workers) and supplementary for the rest (mainly informal sector workers).^37^ PHI cover about 9% of the insured Kenyan population while the remainder are covered by employee insurance schemes.^33^ Additional respondents were identified through snowballing including research organizations, development agencies, labour union representatives, and lobby groups representing insurers, providers and consumers. The interviews were conducted by KM and SM, and lasted about 40 minutes each at locations convenient to the interviewees. The interviews were audio recorded and supplemented by note taking. Interviews were conducted between April 2014 and May 2015. Table 1 shows the summary of respondents.

**Table 1:** Summary of Respondents

**Respondent**	**Number Interviewed**
NHIF officials	4
MoH officials	7
Private health insurance officials	13
CBHI officials	17
Insurance regulators	2
Development agencies and research organizations	3
Independent actuaries and consultants	2
Insurance industry association/lobby group	2
Health service providers	9
Provide regulator	1
Civil society	2

Abbreviations: NHIF, National Hospital Insurance Fund; CBHI, community-based health insurance; MoH, Ministry of Health.

### Conceptual Framework

We applied a conceptual framework developed by Figueras et al^9^ based on agency theory,^38^ and further developed by the Responsive and Resilient Health Systems (RESYST) Consortium.^7^ In agency theory,^38^ the principal (the under-informed party) utilises incentives to induce the agent (the informed party) to act in a way that benefits the principal based on the assumption that the principal can predict how the agent will respond to any particular incentive, and that there is an institutional framework which ensures that the principal will keep their end of the bargain. This implies that the principal can take specific actions to ensure certain outcomes: a relationship applicable to the concept of strategic purchasing.

The conceptual framework of Figueras et al^9^ identifies three pairs of principal-agent relationships: government-purchaser, purchaser-provider, and citizen-purchaser. The RESYST Consortium^7^ further unpacked these relationships into specific actions that should be undertaken to achieve strategic purchasing (Table 2). The framework examined the presence or absence of these actions at policy level (to identify policy gaps) and in practice (to identify practice gaps) based on objective judgment criteria but without specifying any pre-determined indicator. This study examined the purchasing practices of the NHIF and compared them with the theoretical ideal proposed by the RESYST framework for strategic purchasing. Only part of the framework and related results are presented in this manuscript to highlight key findings with more details published elsewhere.^7,37^

**Table 2:** Strategic Purchasing Actions

**Strategic purchasing actions for the Government-Purchaser relationship**
• The government should have policy and legislative frameworks for purchasing activities
• The government should ensure the accountability of purchasing organizations
**Strategic actions for the Purchaser-Provider relationship**
• The purchaser should choose and contract health service providers based on their capabilities including the services offered and their geographical location
• The purchaser should establish mechanisms to ensure service quality including formularies, standard treatment guidelines and essential drug lists
• The purchaser should utilise provider payment mechanisms to incentivise efficiency, service quality and promote equitable access
**Strategic purchasing actions for the Citizen-Purchaser relationship**
• The purchaser should develop benefit packages based on an assessment of the needs, preferences and priorities of the target population
• The purchaser should establish mechanisms to obtain and respond to complaints and feedback from the population

### Data Analysis

Data extracted from document review were summarised using standardised forms. Audio recordings were transcribed verbatim and translated to English, where necessary. Transcripts were entered into QSR NVivo 10 for analysis using a thematic framework approach. Two researchers working independently coded the data, which were charted and categorized into themes derived from the conceptual framework. The charted data were interpreted by identifying and explaining the relationship between key concepts, relating this to theoretical assumptions and identifying policy-relevant messages.

## Results

### Government-Purchaser Relationship

#### Weak Regulatory and Policy Framework

The NHIF is governed by the NHIF Act of 1998,^19^ which outlines the mandate and functions of the NHIF. Statutes related to the conduct of public corporations and public servants also govern the NHIF.^25,39,40^ However, these statutes were silent on strategic purchasing practices. For example, as per strategic purchasing ideals, purchasing organizations ought to develop a benefit package while taking into consideration population needs, national priorities, and evidence of cost-effectiveness. Another key task of purchasers is to undertake activities that enhance health system efficiency and quality of health services. The NHIF Act does not explicitly address these issues even though the Kenyan Constitution emphasizes the importance of equity and effectiveness as central to meeting its citizens’ needs.

The Kenyan health sector is broadly guided by a long-term policy, the Kenya Health Policy (KHP) 2014-2030, and a 5-year strategic plan, the Kenya Health Sector Strategic Plan (KHSSP). The policy and strategic plan attempt to address strategic purchasing, by highlighting the need to improve health systems efficiency and quality. They also prescribe a benefit package of services that should be provided to Kenyans: KEPH.^28^ However, there is no explicit linkage between the policy prescriptions and what the NHIF does in practice. For instance, the NHIF’s benefit package was not guided by KEPH. We illustrate this in Table 3 which shows the list of KEPH services, those provided by the NHIF and a list of the top ten causes of disease according the Kenya Health Policy.

**Table 3:** Summary of NHIF Benefit Package, Kenya Essential Package for Health and Top 10 Leading Causes of Mortality in Kenya

**NHIF National Scheme Benefit Package** ^41,42^	**Kenya Essential Package for Health 2015** ^28^	**Top 10 Causes of Mortality in Kenya in 2009** ^43^
Inpatient benefits depend on category of hospital^a^ • Consultation • Hospital daily charges • Nursing care • Prescribed diagnostic laboratory or other medically necessary services • Physician's, surgeon's, anaesthetist’s or physiotherapist's fees • Operating theatre charges • Specialist consultations or visits • Prescribed drugs/medications and dressings Maternity benefits • Consultation and treatment for both mother and child • Child birth including caesarean section deliveries • Family planning services Outpatient benefits^b^ • General consultation • Diagnostics and treatment of common ailments • Laboratory and investigation • Dental services • Prescribed drugs administration and dispensing • Management of chronic ailments (HIV/AIDS, diabetes, asthma, hypertension, cancer) • Health and wellness education/healthcare counselling such as Screening for conditions eg, cervical and prostate cancer • Treatment of sexually transmitted diseases • Radiology services • Family planning/midwifery/ante/post-natal services • Physiotherapy services • Referral for specialized services	• Immunization • Child health • Screening for communicable conditions • Antenatal care • Prevention of mother to child HIV transmission • Integrated vector management • Good hygiene practices • HIV and STI prevention • Port health • Control and prevention of neglected tropical diseases • Community screening for non-communicable diseases • Institutional screening for non-communicable diseases • Workplace health and safety • Food quality and safety • Pre-hospital care	• HIV/AIDS • Conditions arising during the perinatal period • Lower respiratory infections • Tuberculosis • Diarrhoeal diseases • Malaria • Cerebrovascular disease • Ischemic heart disease • Road traffic accidents • Violence

Abbreviations: NHIF, National Hospital Insurance Fund; STI, Sexually transmitted infection. ^a^ Category A: comprehensive cover for all diseases; Category B: comprehensive cover for all diseases though co-pay required for surgical procedures; Category C: per diem payment for each night of admission. ^b^ The study was conducted before the introduction of outpatient benefits.

This table illustrates that cerebrovascular disease and ischemic heart disease were among the leading causes of death in Kenya in 2009, data on which the KHP was developed, with more recent research supporting this finding.^44,45^ While KEPH lists screening for non-communicable diseases using blood sugar testing, routine blood pressure and body mass index measurement for adults as key intervention,^28^ this was not included in the NHIF benefit package at the time of the study but now is. The new benefit package still omits key interventions such as immunizations and lacked specificity on the interventions covered for example under cervical cancer screening. The linkage of inpatient benefits to the category of hospital means that access to essential surgical services for example following a road traffic accident may be constrained.

Additionally, while the NHIF contracts both public and private healthcare providers, KEPH is public sector and supply side oriented, which makes it difficult to translate to the private sector.^26^ As illustrated in the table above, KEPH contains health promotion and prevention interventions such as port health and mass deworming which are usually supplied through the public sector. This public sector orientation is also identifiable in the KHP which delineates a health system architecture that is couched in public sector terms eg, hospital levels; while not providing a linkage with purchasing mechanisms including the NHIF.^43^ For example the KHP does not mention the NHIF at all, while the KHSSP limits its mention of the NHIF to specifying its mandate to “provide quality social health insurance” (Table 48, p. 71).^27^

The weakness in regulatory and policy framework may be contributed to by a lack of stewardship. Data from interviews with the MoH suggested a lack of clarity about the role of the NHIF, how it should be regulated and whether or not it should enjoy the monopoly of being the sole health insurer with mandatory membership of formal sector workers:

*“…I think there are indications from partners, stakeholders suggesting that we need more than one pool so that people can have a choice, you can choose to join NHIF or another purchaser of your choice…”* [KII_07_MoH].

#### Weak Accountability Mechanisms

The MoH lacked clear structures to provide oversight of the NHIF. While in policy the chief executive officer (CEO) and the board of directors were meant to report to the MoH,^19^ it was unclear how this supported or encouraged strategic purchasing practice. The implication was that monitoring reports did not explicitly address purchasing arrangements, progress, success and challenges. In practice, there were no performance monitoring reports in the public domain and open to scrutiny for this paper, and, more significantly, by citizens.

The NHIF’s accountability framework was also undermined by the absence of a regulatory and policy framework in support of strategic purchasing practice. For instance, the NHIF was accountable to citizens and government through a number of institutions including the MoH, the State Corporations Advisory Committee, the National Treasury, the Kenya National Audit Office, the Inspector General of Corporations, the Efficiency Monitoring Unit and various parliamentary committees. These mechanisms were meant to ensure that the NHIF worked transparently, and that the member contributions were properly utilised.^19^ For example, the NHIF was required to maintain books and prepare financial statements listing income, expenditure, assets and liabilities.^19^ In practice, accountability seemed to be more concerned with financial performance than with other aspects of purchasing activities such as quality of services received by members or responsiveness of the NHIF to complaints.

#### Inadequate Resources Mobilized to Meet Service Requirements

The NHIF draws its revenues from premium contributions, which, as highlighted above, had remained low owing to the failure to revise the rates regularly. This failure resulted from a policy gap that does not address the process for rate revision.^46-49^ In addition, the reliance on voluntary contributions means that NHIF coverage has remained low among informal sector workers: 15% in 2015 even though they accounted for 82% of the Kenyan workforce.^50^

### Purchaser-Provider Relationship

#### The Selection of Providers Compromises Equity

The process for contracting hospitals is guided by the NHIF accreditation regulations of 2003.^19^ In practice the NHIF contracting process involved four steps: application for accreditation, inspection, gazettement and contract signing. Facilities that applied to be accredited were inspected against the NHIF’s accreditation manual which included a checklist that assessed the availability of infrastructure, facilities, equipment, staff and services such as ambulances.^51^ The meaning of accreditation as used by the NHIF is more in keeping with contracting rather than a process by which a health provider, usually an organization, is assessed against a standard developed by an entity separate from that which is being assessed.^52^ The NHIF board of directors acted on the recommendations of the inspection and gazetted the hospital. The contract signed between the NHIF and the health facility specified the category of the health facility, payment mechanisms and rates, and other terms of engagement.

The NHIF contracting process undermined equity. It was reported that the rigorous ‘accreditation’ disadvantaged some regions, which were historically marginalized, thus undermining geographical access. However, there was documentary evidence of de-gazettement (effectively contract cancellation) of health facilities including publicly owned ones for not adhering to NHIF standards.

*“…Imagine the only public hospital in say West Pokot [6th most marginalised county in Kenya with historical underinvestment in social services and amenities], just because it doesn’t meet the standards you say you are not going to accredit that hospital… and for sure you know that members go there… It’s quite an issue, for private not so bad, but for public...”* [KII_13_NHIF].

#### Inadequate Quality Assurance Mechanisms

The NHIF’s quality assurance mechanisms included the use of pre-contracting accreditation, contractual specification, regular inspection, complaints and feedback handling, and the advancement of loans to facilities to improve services. Contractual documents lacked elements that were critical to influencing provider behaviour to the benefit of the purchaser. For example, in the sample contract we had access to, the NHIF relied on facilities to utilise standards and treatment guidelines provided by the MoH even though evidence from Kenyan hospitals suggests there is poor adherence to passively provided guidance.^53-58^

The NHIF was required by law to regularly inspect contracted facilities annually and to continuously monitor adherence to the standards of care established during its initial inspection. A benefits and quality assurance management committee and organizational department existed to oversee quality aspects of services provided by contracted facilities and act as the link between the consumer and the insurer. However this did not always happen as the compliance officers largely engaged with employers and rarely interacted with the beneficiaries. Data from the interviews suggested that the NHIF’s ability to continuously monitor standards or quality of services was limited:

*“No they don’t, what happens with them is that once you have a license from the board then they assume that everything is OK…”* [KII_20_provider].

This was despite the fact that the NHIF had a well functioning information system, branch network and organizational structure that could have supported monitoring activities. This suggests that the focus on the organization was mainly on financial propriety: as demonstrated by the recruitment of medically trained personnel in the claims checking department.

*“I think some time in 1995 there was so much fraud I mean now claims were being manufactured. And because there were no technical people to take care of that, they wouldn’t know that actually this is… It was that bad, so that’s the time the then management decided okay then we need people at least can understand what’s happening”* [KII_13_NHIF].

The NHIF Act allowed the advancement of funds to health facilities to enable them procure essential medical equipment, and in so doing improve quality of services offered to beneficiaries. While the process by which this occurred in practice was unclear, evidence from interviews suggested that some hospitals had benefited from the funding.

*“Yeah, upon approval by our board we can lend a facility funds and we have done that to Moi Teaching Referral [Hospital]. We bought a couple of renal dialysis machines for the big facilities”* [KII_29_NHIF].

#### Poorly Implemented Provider Payment Mechanisms

The NHIF board was mandated by the NHIF Act to determine provider payment rates based on ownership and the accreditation assessment score with the main provider payment mechanisms in use being a per-diem rate for inpatient services. The new outpatient benefit package was paid for using capitation, case-based payments and fee for service for specific services such as renal dialysis and radiology services respectively. The rationale for the overall provider payment system design and the way payment rates were determined was unclear. For example, the choice of capitation was guided in part by the likelihood of over-servicing that theoretically results from fee for service and per diem payments.^14^ While evidence from interviews suggested the use of costing studies and actuarial analysis of NHIF utilisation data in developing capitation rates, these analyses were not in the public domain and it was unclear whether their development included stakeholders. The result was that providers, particularly private ones, were reluctant to be contracted to provide services to the NHIF owing to the low capitation rate which they felt could not cover the cost of care.^59,60^

*“Capitation has been attempted a few times…but it has really not picked up properly…because a lot of times somebody says we are doing an actuarial study but they have got vested interests, so the result is already skewed to favour what you want to come out of it”* [KII_21_provider].

The NHIF had a number of measures to manage unintended consequences of these incentives including regular physical visits to health facilities to ensure that providers did not overcharge; enforced maximum limits on claims payable; fraud training of all staff; and, institutionalization of risk and investigation departments. The NHIF closely monitored claims and made changes to the benefits and quality assurance department to include staff with medical backgrounds to ensure this was done well. However, interviews with providers suggested that physical visits to facilities were the exception, not the norm.

*“No, for the time I have been here, I have never met [NHIF staff] and I’m always in the wards, checking processes…”* [KII_24_provider].

### Citizen-Purchaser Relationship

#### The Development of the NHIF Benefit Package Is not Linked to Needs Assessment, and the Preferences of its Members

The responsibility of designing the benefit package lies with the NHIF’s board of directors. The benefit package was broadly outlined in the NHIF Act, as both outpatient and inpatient services. There was no explicit mention in policy or statute on how needs assessment for NHIF beneficiaries ought to be done. However, the NHIF board was required to protect the interests of contributors, including regulating contribution rates and benefit package. In practice, the NHIF used a variety of means to determine health needs of the population and inform the design of the benefit package, including customer satisfaction surveys; feedback received from board members and analysis of claims data. However, no formal needs assessment activities were undertaken and it was recognized that citizen engagement required improvement.

To promote awareness of the benefit package and the accredited providers, the NHIF published detailed information about the benefit package and providers on the website and advertised widely in the media. Evidence from interviews with citizens who were also members of CBHI schemes suggested dissatisfaction with benefit package contents, information on access to providers and cover for extended families and indigents within the community.

*“Those people who contribute to the NHIF through the [CBHI] schemes are the ones who benefit the most because they know which hospitals are good and which hospitals are bad. Those others who have deductions made from their salaries are taken here and there; no one tells you which hospital to go to”* [CBHI_01].

*“Yeah actually we accommodate because the more members the more money…and it’s a challenge to the NHIF; there they have a maximum, but with us we can accommodate more members, more families. You can even take an orphan to bring into your family…so the community realizes it’s a good move, because you can take somebody’s care even though you are not related”* [CBHI_15].

A related area of concern was the large proportion of operating expenses taken up by administrative expenses (Table 4). These were perceived as being high by stakeholders^46,61^ because this meant that a significant proportion of resources were spent on non-claim settling activities. The NHIF believed that the proportion of administrative expenses would decline over time, largely due to the revised contribution rates which were nearly 5-fold those previously charged.^62^ More up-to-date financial statements were not available to the authors for assessment, and the financial statements accessed in 2015 have since been removed from the NHIF website.^63^ However, the contribution figures are corroborated by those reported in other government documents such as the Economic Survey.^50^

**Table 4:** Comparing NHIF Contributions and Operating Expenses

	**2014**	**2013**	**2012**^a^	**2011**
	**KES '000**^b^	**KES '000**	**KES '000**	**KES '000**
Contributions (national scheme and civil servants and disciplined forces medical scheme)	13 629 140	12 229 966	9 595 592	6 628 729
Operating expenses less benefit expenses	3 982 803	3 527 918	3 287 600	2 780 489
Operating expenses as a percentage of contributions	29%	29%	34%	42%

Abbreviation: NHIF, National Hospital Insurance Fund. ^a^ Start of Civil Servants and Disciplined Forces Medical Scheme. ^b^ KES: Thousands of Kenya Shillings ie, the first cell would be 13.629 Billion. Source: Authors’ calculations based on NHIF Financial statements (2012-2014).

*“But then people fail to understand one thing, that we have had contribution rates stuck since 1989 about over twenty-something years now…all along NHIF has been expanding, growing...When we got this civil servants scheme, we brought down that percentage to 28 [%] without doing anything…just having them entering as revenue and going out as whole payments to benefits...”* [KII_29_NHIF].

Though the NHIF’s benefit package was fairly comprehensive, its measures for protecting beneficiaries from the financial burden of ill health were likely to be ineffective. First, the payment rates for capitation and inpatient reimbursements were perceived to be low by providers.^64^ This led to balance billing by healthcare providers. Second, the NHIF cap on benefits was at 180 days of admission. Third, members incurred penalties for late payment of premiums. Previously five times the unpaid up amount, these penalties had been reduced in 2014 so that informal sector members, who made voluntary premium contributions, were required to pay 25% of the amount defaulted as penalties while formal sector members were required to pay a penalty of two times the unpaid amount.^65^ Finally, the NHIF covered a small proportion of the population and excluded non-contributors who were more likely to be the poor.^20^ Moreover, retaining voluntary members was a challenge. This segment of members, made up mostly of informal sector workers, tended to take up insurance only when they were ill, and would allow their membership to lapse especially when they had not made claims that year, or when they realized that they could not access outpatient benefits. The application of penalties for failure to keep up with premium payments was also cited as affecting retention.

*“They will come in, you will only get the sick ones most of the times and when they come in they come with the intention of accessing the benefits. And when they access the benefits many of them do not continue when they come. So if you look at the claims ratio for the informal sector vis-à-vis the other sectors it’s very high”* [ KII_30_NHIF].

To cater for this, the NHIF had made administrative arrangements allowing members to either make up the missed payments within 5 days or start over again with a 60-day exclusion period.

#### Inadequate Complaints and Feedback Mechanisms

The NHIF Act does not provide for feedback or complaints mechanism for beneficiaries or members. However, the board of directors was composed of key stakeholders including labour unions, while provisions of the public officer ethics act covered some of these concerns.^40^ For example, the NHIF employees were required to seek to improve the performance of their organization and give honest and impartial advice to all.

The NHIF website provided an email address and phone contact for use by beneficiaries. Newspaper advertisements, and interviews, specified that the phone line was toll free and operated 24 hours a day. Our attempts to access the toll-free number during the study period were unsuccessful. The NHIF’s use of its website, newspapers and media pronouncements to inform the populace of its service entitlements limited the reach of its messages to those who had access to these media. Furthermore, there was no public forum for reporting performance.

While there was evidence that these feedback mechanisms did work, for example resulting in the redesign of the enrolment form, it was unclear what processes were in place to regularly incorporate this feedback in benefit package design and other aspects of purchasing performance. While interviews with NHIF officials suggested that changes to the benefit package and premium rates were based on member feedback, the process of implementation of these changes was met with stiff opposition from labour unions and the general population.^48,49^

## Discussion

The NHIF remains the main purchaser of healthcare services for the insured in Kenya. While several efforts have been directed towards improving its purchasing arrangements, there are shortcomings when examined through the lens of ideal strategic purchasing practice. These shortcomings were identifiable along all three principal-agent axes.

Policy and regulatory frameworks are essential to strategic purchasing practice. For example, Ghana’s UHC reforms of the National Health Insurance Scheme, is based on series of policy and legal structures, the most recent of which has faced challenges after a period of initial success in improving coverage.^66^ However, policy implementation requires specific attributes such as planning and negotiating skills, which are absent in many health system practitioners in LMICs.^67,68^ This implies that both areas ie, policy development and policy implementation are key targets for ensuring the adoption of strategic purchasing practice. Kenya’s decentralised context presents an additional if surmountable challenge to the policy development and implementation process. Decentralisation can result in a policy and practice impasse particularly where reforms are a consequence of broader reforms.^69,70^

The absence of a policy and regulatory framework for strategic purchasing in Kenya was exacerbated by a lack of clarity at the MoH about the centrality of the NHIF to health financing reforms. This had the potential to deny the NHIF the advantages of monopsony power. This power is particularly useful in directing provider action for example in negotiating price or even service delivery structure. In Thailand, for example, the National Health Security Office (NHSO) drove the development of primary care networks which have increased access to services and allowed for better management of financial resources.^71^ As such, the lack of stewardship from the MoH undermined strategic purchasing action of the NHIF.

This study was not set up to examine the link between organisational structures and institutional arrangements of the NHIF and its purchasing actions. However, previous assessments of the NHIF have recognised the need to address issues such as the governance and accountability arrangements in the organization.^61^ Recent reforms have sought to alter the governance structure of the NHIF for example through changes to the board and to the recruitment process for the chief executive officer.^65^ Future research should explore the effects of these changes and the potential for further alignment of the NHIF as an organization towards strategic purchasing practice.

Quality of care is a complex issue with limited evidence of what works in practice.^58,72^ Nevertheless, literature suggests a number of policy levers that could be utilised by purchasers to influence quality and efficiency of the health system.^73^ Strategic purchasing offers an approach that would enable the implementation of quality improvement strategies using a whole systems perspective.^74^ Underlying the relationship between the purchaser and provider is the contract whose key elements should include the type of service to be delivered, specification of performance requirements, its duration and proposed payment mechanism.^9,75,76^ As the findings indicate, the NHIF’s accreditation and contracting process, while well intended is very infrastructure oriented and does not address process and outcome aspects of quality of care. Contract enforcement remains a challenge, while other key elements are not comprehensively addressed. In Thailand’s Universal Coverage scheme, the contract specifies the contracting unit, catchment area for primary care facilities as well as provider payment mechanisms.^71,77^ On the other hand, rigid contracting mechanisms contributed to the lack of strategic purchasing observed in neighbouring Vietnam.^78^ In Nigeria, the complex contractual relationship between providers, health management organizations and the National Health Insurance Scheme weakened strategic purchasing practice in the Formal Sector Social Health Insurance Programme.^79^

The NHIF inpatient-only benefit package favoured hospitals, at the expense of lower end facilities that were predominantly used by the poor.^80^ This arrangement had the potential to promote inequities and inefficiency for example by promoting hospital utilisation for illnesses that could be managed at primary care level. Strategic purchasing offers the opportunity to correct the misdirection of care that favours curative services at the expense of preventive and promotive services.^81^ Strategic purchasing can address equity in hospital care by selecting providers in a way that expands geographic access,^7^ and set priorities for hospital services in relation to social and public health priorities.^82^ Hospital efficiency can also be improved through better alignment of referral systems, integration of care, selection of cost-effective services, and specialisation.^82^

Overall, the effect of different provider payment mechanisms on the healthcare system depends on their design, how their mix reflects the local context and how well they are implemented.^13,14^ Our findings suggest shortcomings in all three of these aspects. Besides the lack of information on how the payment systems were designed or rates were arrived at, this significant health financing reform lacked the required buy in from the various stakeholders including providers.^83^ Moreover, these changes did little to address key contextual issues such as the power of providers over purchasers which limited strategic purchasing actions. This power was the result of a limited supply of providers in quality, quantity and spread; better lobbying and negotiating ability; multiplicity of revenue sources for providers including out of pocket payments; and provider control of key processes such as licensing and price setting. In addition, the continued use of fee for service, for example, opened the health system to over servicing and other forms of inefficiency in the absence of clear goals to increase access to certain services.^1,9,14^ There were no mechanisms in place at the time of the study to deal with the potential shortcomings of capitation which can lead to reduced provision of services and increased referral to higher levels of care.^14^ With evidence of weak monitoring of service delivery, there is little to guarantee that these unintended consequences are not already present among NHIF contracted providers.

The results have shown that the link between the beneficiaries and the NHIF is weak. This manifests itself in various ways including in a benefit package that deviates not only from policy design for access to care but also from burden of disease patterns as illustrated in this paper. Following on from the example of screening for non-communicable disease, the newly introduced outpatient benefit package is still silent on the specific interventions that fall under this service category.^41^ The lack of this linkage potentially undermines progress to UHC, and to the attainment of wider societal goals such as health standard maximisation.^43^ This disconnect is all the more important in a country that is considering mobilizing resources through mandatory contributory health insurance. Limited citizen engagement may undermine not only premium contributions but also willingness to contribute to the NHIF through tax revenue: a key source of domestic financing essential for UHC.^84,85^ Equity in utilization and financial risk protection are similarly undermined by the NHIF’s action and inaction. Segmenting the population, limiting coverage to contributors and penalties for non-payment result in a barrier to access to financial risk protection and to health services. Besides low NHIF coverage in the informal sector, benefit incidence analysis suggests a predominantly pro-rich distribution of health service benefits.^86^ Evidence from an assessment of effective coverage confirms the inequitable receipt of effective health services.^87^ As such, the NHIF may inadvertently exacerbate existing inequalities and risk of financial catastrophe and so deviate from strategic purchasing’s pursuit of UHC goals.

The findings presented in this paper offer lessons for policy makers in other LMICs. First, it highlights the need to develop and implement a strategic purchasing framework. This framework would necessarily be embedded in a wider health financing strategy and health sector regulatory framework that identifies clearly with local contexts. Included in this framework would be key provisions that would address the shortcomings on the three principal-agent relationships. On the government-purchaser axis, the framework should support the development and implementation of policy and regulations that support strategic purchasing. Needs assessment and elicitation of feedback and complaints, on the citizen-purchaser axis, should be formalised with clear mechanisms for including their findings in benefit package design. Moreover, benefit packages should offer sufficient financial risk protection by being universally available, with regulations on balance billing and with explicit exclusions that consider equity. Contracts with providers should encourage quality and efficiency by specifying key actions and conditions. These would include the selection and use of evidence-based treatment guidelines and essential drug lists by providers, the utilisation of an optimal mix of provider payment mechanisms, and the use of information to monitor provider performance. These contracts would need to be supported by policy that give purchasers room to chose who they contract with, provider payment mechanisms and payment rates.

## Conclusion

While this study examined purchasing arrangements of the NHIF, we appreciate that this is a partial analysis, and that a health system performance is influence by the interaction between all health financing functions. Nonetheless, our analysis offers important insights into an often neglected health financing function, purchasing, and specifically the actions of a key purchasing agent in Kenya, the NHIF. Addressing the gaps in the NHIF’s purchasing performance requires a number of approaches. Critically, there is a need for the government through the MoH to embrace its stewardship role in health, while recognizing the multiplicity of actors given Kenya’s devolved context. Relatively recent decentralisation reforms present an opportunity that should be grasped to rewrite the contract between the government, the NHIF and Kenyans in the pursuit of UHC.

Specifically, we recommend the development of a policy and regulatory framework for strategic purchasing practices. This would lay the ground for strategic purchasing practice for the NHIF that would be inclusive, coherent and contextual. Inclusiveness would mean including stakeholders where needed, coherence would mean fitting with wider policy while context would mean accounting for current decentralised arrangements and labour market structure. This framework would also address our second key recommendation for a person-centred and evidence-based benefit package. Given recent reforms surrounding the benefit package, our recommendation is that the NHIF’s new package should be critically examined to a whether it reflects the needs, preferences and values of Kenyan citizens and fits with national priorities and goals. Our final recommendation concerns ensuring the quality of care and the appropriate use of incentives to guide provider behaviour in pursuit of this goal. We propose that the NHIF take urgent measures to review its quality assurance mechanisms, and in particular consider the incentives generated by its mix of provider payment mechanisms, monitoring capabilities and contractual requirements.

## Acknowledgements

This manuscript is published with the permission of the Director of KEMRI. Funds from the Wellcome Trust (#101082) awarded to JC supported KM and SM. EB is funded by a Wellcome Trust Research Training Fellowship (#107527). Additional funds from a Wellcome Trust core grant awarded to the KEMRI-Wellcome Trust Research Program (#092654) supported this work. The funders and the World Bank had no role in study design, data analysis, decision to publish, drafting or submission of the manuscript. The views expressed in the papers are for the authors and not for the organizations they represent.

## Ethical issues

The KEMRI Scientific and Ethics Review Unit approved this study under KEMRI SSC No. 2795. Written informed consent was obtained from all participants with permission sought from institutional and organizational heads.

## Competing interests

Authors declare that they have no competing interests.

## Authors’ contributions

JC conceptualised the study. KM and SM collected data and performed preliminary analysis. KM developed the first draft. All authors contributed to subsequent and final drafts as well as further analysis.

## Authors’ affiliations

^1^Health Economics Research Unit, KEMRI Wellcome Trust Research Programme, Nairobi, Kenya. ^2^Kenya Country Office, The World Bank, Nairobi, Kenya.

## Key messages

**Implications for policy makers**

- Strategic purchasing requires a policy and regulatory framework that supports, among other things, person-centred evidence-based benefit package design.
- Well intentioned policy and actions can inadvertently exacerbate health inequalities, inefficiency and poor quality.
- Strategic purchasing requires capacity in purchasing organizations in contracting, provider payment mechanisms design and implementation, and monitoring.

**Implications for public**

Improving the way in which funds for health are used for the purpose of obtaining health services is important in making sure every person can access health services without suffering financial hardship. Using Kenya’s National Hospital Insurance Fund (NHIF) as an example, we highlight gaps in policy and practice in how funds for health are used. We also provide lessons for health stakeholders in Kenya and similar settings. Implementing these recommendations may improve policy and practice and ensure access to needed health services of good quality by all citizens without imposing unnecessary financial burden.
